# In Vivo Antimicrobial Activity of Nisin Z Against *S. aureus* and Polyurea Pharmadendrimer PURE_G4_OEI_48_ Against *P. aeruginosa* from Diabetic Foot Infections

**DOI:** 10.3390/antibiotics14050444

**Published:** 2025-04-28

**Authors:** Isa Serrano, Dalila Mil-Homens, Rita F. Pires, Vasco D. B. Bonifácio, Joana F. Guerreiro, Eva Cunha, Sofia S. Costa, Luís Tavares, Manuela Oliveira

**Affiliations:** 1 CIISA—Center for Interdisciplinary Research in Animal Health, Faculty of Veterinary Medicine, University of Lisbon, Av. Universidade Técnica, 1300-477 Lisbon, Portugal; jguerreiro@fmv.ulisboa.pt (J.F.G.); evacunha@fmv.ulisboa.pt (E.C.); ltavares@fmv.ulisboa.pt (L.T.); moliveira@fmv.ulisboa.pt (M.O.); 2 Associate Laboratory for Animal and Veterinary Sciences (AL4AnimalS), Faculty of Veterinary Medicine, University of Lisbon, Av. Universidade Técnica, 1300-477 Lisbon, Portugal; 3 iBB—Institute for Bioengineering and Biosciences and i4HB—Institute for Health and Bioeconomy, Instituto Superior Técnico, University of Lisbon, Av. Rovisco Pais, 1049-001 Lisbon, Portugal; dalilamil-homens@tecnico.ulisboa.pt (D.M.-H.); ritafpires@tecnico.ulisboa.pt (R.F.P.); vasco.bonifacio@tecnico.ulisboa.pt (V.D.B.B.); 4 Bioengineering Department, Instituto Superior Técnico, University of Lisbon, Av. Rovisco Pais, 1049-001 Lisbon, Portugal; 5 Global Health and Tropical Medicine (GHTM), Associate Laboratory in Translation and Innovation Towards Global Health (LA-REAL), Instituto de Higiene e Medicina Tropical (IHMT), Universidade Nova de Lisboa (UNL), R. da Junqueira 100, 1349-008 Lisbon, Portugal; scosta@ihmt.unl.pt; 6 cE3c—Centre for Ecology, Evolution and Environmental Changes & CHANGE—Global Change and Sustainability Institute, Faculty of Sciences, University of Lisbon, Campo Grande 016, 1749-016 Lisbon, Portugal

**Keywords:** diabetic foot infection, Nisin Z, core–shell polycationic polyurea pharmadendrimer, Amlodipine, *Galleria mellonella*, *Pseudomonas aeruginosa*, *Staphylococcus aureus*

## Abstract

Background/Objectives: Diabetic foot infections (DFIs) are commonly associated with frequent hospitalizations, limb amputations, and premature death due to the profile of the bacteria infecting foot ulcers. DFIs are generally colonized by a polymicrobial net of bacteria that grows in biofilms, developing an increased antimicrobial resistance to multiple antibiotics. DFI treatment is a hurdle, and the need to develop new therapies that do not promote resistance is urgent. Therefore, the antibacterial efficacy of Nisin Z (antimicrobial peptide), a core–shell polycationic polyurea pharmadendrimer (PURE_G4_OEI_48_) (antimicrobial polymer), and amlodipine (antihypertensive drug) was evaluated against *S. aureus* and *P. aeruginosa* isolated from a DFI and previously characterized. Methods: The antibacterial activity was analyzed in vitro by determining the minimal inhibitory concentration (MIC) and in vivo in a *Galleria mellonella* model by assessing the larvae survival and health index. Results: The results indicate that Nisin Z exhibited antibacterial activity against *S. aureus* in vivo, allowing larvae full survival, and no antibacterial activity against *P. aeruginosa*. Nisin Z may have reduced the antibacterial effectiveness of both PURE_G4_OEI_48_ and amlodipine. PURE_G4_OEI_48_ significantly increased the survival of the larvae infected with *P. aeruginosa*, while amlodipine showed no activity against both bacteria in vivo. Conclusions: These findings suggest that both Nisin Z and PURE_G4_OEI_48_ could potentially be used individually as adjunct treatments for mild DFIs. However, further studies are needed to confirm these findings and assess the potential toxicity and efficacy of PURE_G4_OEI_48_ in more complex models.

## 1. Introduction

Diabetes mellitus is a chronic metabolic disease that affects 537 million people worldwide, a number that is expected to double by 2030 [1,2]. It compromises the patients’ vascular and immune systems, leading to nerve damage in the legs and feet [3]. As a consequence, diabetic foot ulcers (DFUs) may develop in about 30% of cases [4], and 50% of DFU patients suffer diabetic foot infections (DFIs) due to skin barrier loss [4], with around two-thirds of chronic foot infections being polymicrobial [5,6]. Infected ulcers are strongly linked to a reduced quality of life, frequent hospitalizations, lower extremity amputations, and premature death [7].

*Staphylococcus aureus* is the primary DFI pathogen, often found together with Gram-negative species, like *Pseudomonas aeruginosa*, in severe cases [5,8]. Both belong to the ESKAPE group of pathogens and are included on the WHO’s priority list for the development of new antibiotics [6,9,10] due to biofilm formation ability, virulence factors production, and multidrug resistance profiles [5,6,8]. About 25% of severe DFI cases remain unresolved [11] due to difficulties in establishing proper treatments, especially if complex biofilms are present [12]. Broad-spectrum antibiotics are commonly used in these cases [13,14], with topical administration being applied in mild infections [14]. So, the discovery and development of innovative topical therapeutic compounds that could act as antimicrobials while not promoting resistance development is urgent. Within this context, promising compounds include membrane-acting drugs that act independently of the bacteria’s metabolic state, such as antimicrobial peptides (AMPs), like Nisin [15,16,17]; synthetic mimics of antimicrobial peptides (SMAMPs), like core–shell polycationic polyurea pharmadendrimers [18]; and other drugs, such as amlodipine [19,20,21], which were all recently demonstrated as effective against Gram-positive and Gram-negative bacteria.

Nisin, a 34-amino-acid cationic bacteriocin produced by *Lactococcus lactis*, was already described as a promising alternative DFI treatment [22,23,24,25]. While Nisin A targets Gram-positive bacteria, Nisin Z has a broader activity, especially against Gram-negatives [16,17], when combined with EDTA [15,26]. Nisin acts by binding to lipid II, a key peptidoglycan precursor, thereby reducing the chances for resistance development [27,28]. Overall, it has limited direct immunogenicity in humans, but its immunomodulating effects [28,29] and the need for conjugation with carrier proteins to induce antibody production are important factors to consider in its application [30]. Despite their benefits, the therapeutic use of AMPs, like Nisin, face challenges like protease degradation, salt sensitivity, and high production costs [31]. Other potential compounds include polyurea pharmadendrimers, which are synthetic AMP mimics with dual antimicrobial and anticandidal action [18] that act by rapidly disrupting microbial membranes, therefore reducing the risk of resistance development [32]. Moreover, amlodipine, a calcium channel blocker used for the control of hypertension, angina, and diabetes [33], also shows antibacterial activity against *P. aeruginosa* and *S. aureus* [19,20,21]. It may aid with intracellular bacterial killing by targeting macrophages, reducing the risk of drug resistance development [21].

This study aimed to evaluate the potentials of Nisin Z, a core–shell polycationic polyurea pharmadendrimer (PURE_G4_OEI_48_), and amlodipine to be used as topical therapies for treating mild superficial DFIs. Their antibacterial efficacy against two DFI isolates, *S. aureus* Z25.2 and *P. aeruginosa* Z25.1, was evaluated both in vitro and in vivo. In vitro tests focused on the determination of minimal inhibitory concentrations (MICs), while the in vivo studies using a *Galleria mellonella* model assessed the larvae survival rates and health indices. By combining in vitro and in vivo approaches, this study sought to provide a more comprehensive understanding of the therapeutic potential of these compounds against clinically relevant DFI pathogens. The findings could contribute to the development of alternative topical treatments aimed at improving infection control and reducing the risk of antibiotic resistance.

## 2. Results

### 2.1. Minimum Inhibitory Concentration (MIC)

The MIC values for Nisin Z, PURE_G4_OEI_48_, and amlodipine are shown in Table 1.

The Nisin Z MIC (mg/mL) values toward *S. aureus* Z25.2 and *P. aeruginosa* Z25.1 confirmed that *P. aeruginosa* Z25.1 was not inhibited by Nisin Z (Table 1), as previously shown [15]. In this case, 0.4 mg/mL was the highest concentration tested, and no inhibition was observed. The MIC value (mg/mL) of PURE_G4_OEI_48_ was much higher in comparison with the MIC values of Nisin Z and amlodipine toward *S. aureus* and just 3 times higher than amlodipine MIC towards *P. aeruginosa*, reflecting the specific interactions between the drug and the strain, as well as its pharmacokinetics. A low micromolar MIC may indicate stronger activity of Nisin Z and PURE_G4_OEI_48_ against *S. aureus* and *P. aeruginosa*, respectively.

The effect of the supplementation of PURE_G4_OEI_48_ and amlopidine solutions with Nisin Z was also tested using a Nisin Z concentration of 0.2 mg/mL to ensure a higher bacteriostatic and antibiofilm efficiency toward *S. aureus* Z25.2 and *P. aeruginosa* Z25.1 [15]. When supplemented with this concentration of Nisin Z, the MIC values of PURE_G4_OEI_48_ and amlodipine remained unaltered, except for an increase in the PURE_G4_OEI_48_ MIC value against *P. aeruginosa* (2.07 mg/mL).

### 2.2. Galleria Mellonella Killing Assay

For the in vivo experiments, a single concentration each of PURE_G4_OEI_48_ (2.07 mg/mL) and amlodipine (0.16 mg/mL) were tested for both *S. aureus* and *P. aeruginosa*. These concentration values were selected to guarantee the bioavailability of the drugs and their effectiveness in the experimental model. These concentrations would allow for sufficient amounts of the drugs to reach their target bacteria in vivo, making them effective for the intended treatment.

Nisin Z was tested in vivo at 0.2 mg/mL, a concentration that enhances its antibacterial efficiency while remaining safe for pharmaceutical and medical applications [15]. This specific concentration was chosen based on the results from a previous study, which demonstrated that Nisin Z with EDTA (0.4%) provided superior antibacterial activity (MIC ≥ 0.001 ± 0.0011 mg/mL for *S. aureus* Z.25.2 and MIC ≥ 0.0025 mg/mL for *P. aeruginosa* Z.25.1 and dual cultures), enhanced bacteriostatic efficacy (MBC ≥ 0.0086 ± 0.0053 mg/mL for *S. aureus* Z.25.2, MBC ≥ 0.15 ± 0.0535 mg/mL for *P. aeruginosa* Z.25.1, and MBC ≥ 0.1 µg/mL for dual cultures), and greater antibiofilm effectiveness (MBIC ≥ 0.0005 mg/mL for *S. aureus* Z.25.2 and MBIC ≥ 0.0125 ± 0.0071 mg/mL for *P. aeruginosa* Z.25.1) than Nisin Z [15]. The inhibitory potential of Nisin Z plus EDTA 0.4% was tested in vivo, and it was observed that EDTA supplementation did not improve the antibacterial efficacy of Nisin Z against *P. aeruginosa* Z25.1. Therefore, in all further experiments, Nisin Z was tested at the same concentration but without being supplemented with EDTA.

#### 2.2.1. Health Index Score

**Figure 1:**
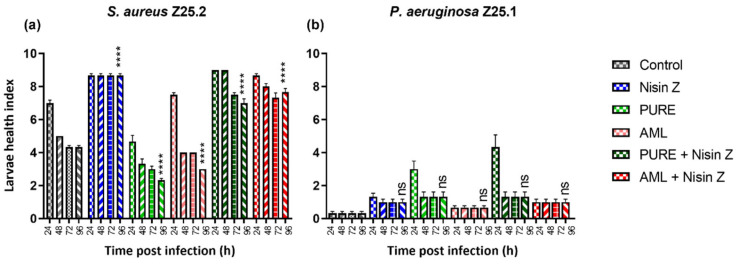
Health index of larvae infected with *S. aureus* Z25.2 (**a**) and with *P. aeruginosa* Z25.1 (**b**) treated with Nisin Z (0.2 mg/mL), PURE_G4_OEI_48_ (2.07 mg/mL) with and without supplementation with Nisin Z, and amlodipine (0.16 mg/mL) with and without supplementation with Nisin Z at 24, 48, 72, and 96 h post-infection. Positive control: infected larvae + NaCl 0.9% (grey bar); negative control: uninfected larvae + NaCl 0.9%/each treatment. Data are shown as the mean ± standard error (larvae activity, cocoon formation, melanization, and survival) of three independent determinations for 10 animals per treatment. ns, not significant; ****, *p* < 0.0001 from the control at 96 h (one-way ANOVA, Tukey’s multiple comparison test). PURE, PURE_G4_OEI_48_; AML, amlodipine.

The health index score (Figure 1), which scores four main parameters: larvae activity, cocoon formation, melanization, and survival [34], was determined. The larvae activity was scored as follows: 3 for movement without stimulation, 2 for movement when stimulated, 1 for minimal movement, and 0 for no movement. Cocoon formation was scored as 1 for a fully formed cocoon, 0.5 for a partially formed cocoon, and 0 for no cocoon formation. Melanization was graded from 4 (no melanization) to 0 (complete melanization, fully black larvae), with intermediate scores based on the number and extent of dark spots (3—less than three spots on beige larvae, 2—≥ three spots on beige larvae, 1—dark spots on brown larvae). Survival was assessed with 2 points for live larvae and 0 for dead larvae [34]. The final score was calculated as the average sum of the parameters for the 10 treated larvae per group, measured in triplicate.

The health index score was higher in larvae infected with *S. aureus* than in those infected with *P. aeruginosa*. At 96 h post-infection with *S. aureus*, the health index scores of the test groups after treatment with Nisin Z, with PURE_G4_OEI_48_ supplemented with Nisin Z, and with amlodipine supplemented with Nisin Z were significantly higher (*p* < 0.0001) than the one of the control group (Figure 1a). In contrast, the health index score of this test group after treatment with PURE_G4_OEI_48_ and amlodipine was significantly inferior (*p* < 0.0001) than the one of the control group (Figure 1a).

Regarding the larvae infected with *P. aeruginosa*, the health index score of this test group was not significantly different from the one of the control group at 96 h after the application of all treatments under evaluation (Figure 1b). These outcomes are likely attributable to the higher toxicity of PURE_G4_OEI_48_ and amlodipine in the larvae infected with *S. aureus*, which may have inhibited the host’s capacity to control the *S. aureus* infections. In contrast, the *P. aeruginosa* infections were unaffected by the applied treatments, implying a different host–pathogen interaction mechanism.

**Figure 2:**
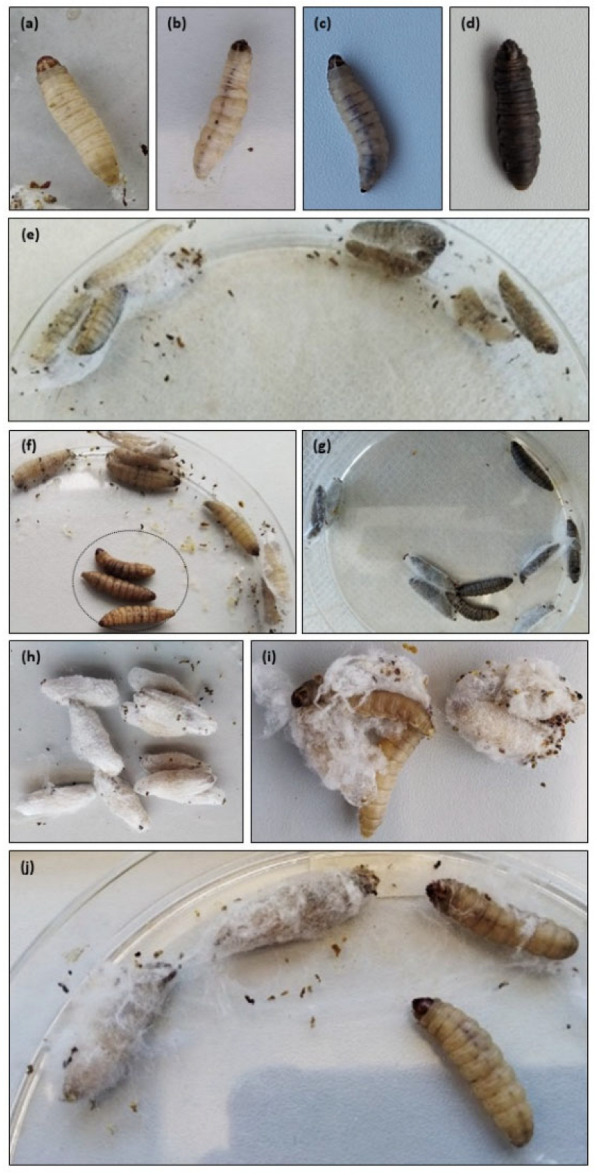
Degrees of larvae melanization: (**a**) no melanization; (**b**) dark spots on cream larvae; (**c**) dark spots on brown larvae; (**d**) full melanization. Larvae melanization at 24 h: (**e**) negative control (saline plus saline) showing no melanization (cream larvae); (**f**) larvae challenged with *S. aureus* showing partial melanization (brown dead larvae, inside the circle) and no melanization (cream live larvae); (**g**) larvae challenged with *P. aeruginosa* showing full melanization (dark dead larvae). Cocoon development at 96 h: (**h**) negative control (saline plus saline) with a full development of the cocoon; (**i**) caterpillars following *P. aeruginosa* infection and PURE_G4_OEI_48_ treatment with a partial and altered cocoon; (**j**) negative control (saline plus PURE_G4_OEI_48_) with a partial and altered cocoon. As shown, PURE_G4_OEI_48_ delayed and affected the normal development of *G. mellonella* cocoons (medium larval length: 2 cm).

Photos representing the larval melanization degrees, larval melanization at 24 h, and cocoon development at 96 h are shown in Figure 2.

The live larvae, including those belonging to the negative control group (see Section 4.4), were characterized by being totally cream (Figure 2a,e). The infected larvae developed some dorsal spots (Figure 2b). As expected, most dead larvae were totally black (Figure 2d,g) or presented some degree of melanization on the body (Figure 2c,f).

All larvae from the negative control group formed full cocoons at 96 h (Figure 2h). Except for the larvae to which PURE_G4_OEI_48_ was administered, all larvae that belonged to the test groups (infected and subjected to treatment) and to the positive control group (infected and not subjected to treatment) that survived formed full cocoons. The larvae to which PURE_G4_OEI_48_ was administered did not form cocoons or showed delayed cocoon formation with an erratic and disordered arrangement (Figure 2i,j).

#### 2.2.2. Survival Curves

The larvae within the negative control group fully survived, which demonstrated that Nisin Z, PURE_G4_OEI_48_, and amlodipine were non-toxic to this insect model.

The lethal dose of each bacterium was previously determined. The lethal dose determination must include the time needed for the bacteria to infect the larvae and for the drug to act. Generally, 100 colony forming units (CFU) of *P. aeruginosa* killed all the larvae between 24 h and 48 h after inoculation, whereas 1 × 10^6^ CFU of *S. aureus* killed 30–40% of the larvae at 72 h after inoculation. The same outcome was observed with 1 × 10^8^ CFU of *S. aureus*, suggesting that a higher larval mortality rate was unlikely. Therefore, a lower CFU of 1 × 10^6^ was used.

**Figure 3:**
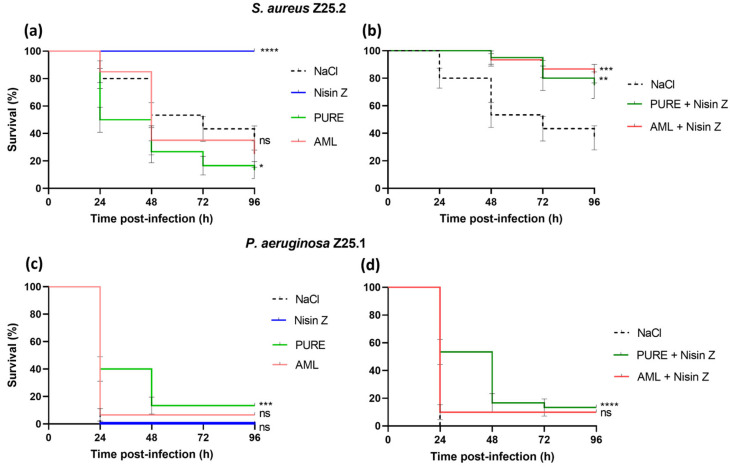
Kaplan–Meier survival curves for *G. mellonella* larvae after infection with *S. aureus* (1 × 10^6^ CFU) and *P. aeruginosa* (100 CFU), either with (full line) or without (dashed line) administration of the compounds under evaluation at 1 h post-infection. (**a**,**c**) Nisin Z (0.2 mg/mL), PURE_G4_OEI_48_ (2.07 mg/mL), and amlodipine (0.16 mg/mL); (**b**,**d**) PURE_G4_OEI_48_ supplemented with Nisin Z and amlodipine supplemented with Nisin Z. Positive control: infected larvae + NaCl 0.9% (dashed line); negative control: uninfected larvae + NaCl 0.9%/treatment. In subfigures (**c**,**d**), the dotted line is present but may not be visible due to overlap with other lines in the figure. The results represent the mean of three independent determinations for 10 animals per treatment. The bars represent the standard error. The Mantel–Cox test significance between survival curves of treated larvae and nontreated larvae: ns, not significant; *, *p* = 0.01; **, *p* = 0.005; ***, *p* = 0.0001; ****, *p* < 0.0001. PURE, PURE_G4_OEI_48_; AML, amlodipine.

Figure 3 shows the Kaplan–Meier survival curves for the *G. mellonella* larvae.

The treatment with Nisin Z led to the full survival of larvae infected with *S. aureus* Z25.2 (*p* < 0.0001) (Figure 3a). When combined with Nisin Z, both PURE_G4_OEI_48_ (*p* = 0.005) and amlodipine (*p* = 0.0001) showed antibacterial activity against *S. aureus*, with the test groups displaying significantly higher larvae survival rates than the one from the control group (Figure 3b). When administered alone, PURE_G4_OEI_48_ and amlodipine showed no antibacterial effect against *S. aureus*. The larvae from the test group treated with these compounds had lower survival rates than those from the control group, with a significant difference observed between the control and PURE_G4_OEI_48_ treatment groups (*p* = 0.01) (Figure 3a).

Moreover, there were statistical differences between the survival rates of the larvae infected with *S. aureus* treated with PURE_G4_OEI_48_ alone and treated with PURE_G4_OEI_48_ supplemented with Nisin Z (*p* < 0.0001), and between the survival rates of larvae infected with *S. aureus* treated with amlodipine alone and treated with amlodipine supplemented with Nisin Z (*p* < 0.0001).

In all the experiments, all the larvae died upon infection with *P. aeruginosa* Z25.1 after 24 h (Figure 3c). Only the larvae treated with PURE_G4_OEI_48_ alone (*p* = 0.0001) or with PURE_G4_OEI_48_ supplemented with Nisin Z (*p* = 0.0001) showed a significant increase in the larvae survival compared with the control group (Figure 3c,d). The test groups treated with Nisin Z and with amlodipine alone or supplemented with Nisin Z showed no significant differences in the larvae survival compared with the control group (Figure 3c,d).

There were no statistical differences between the survival rates of the larvae infected with *P. aeruginosa* and treated with PURE_G4_OEI_48_ alone or supplemented with Nisin Z, nor between the survival rates of the larvae infected with *P. aeruginosa* and treated with amlodipine alone or supplemented with Nisin Z.

**Figure 4:**
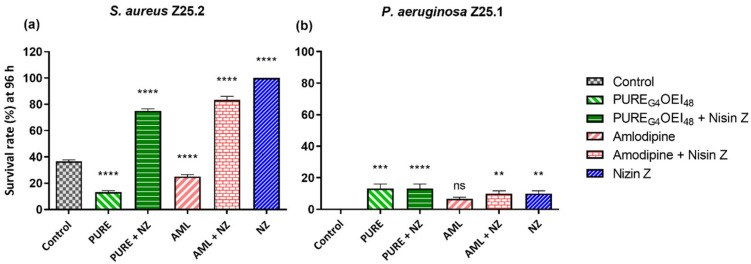
Larvae survival rates (%) at 96 h after challenging them with *S. aureus* Z25.2 (**a**) and with *P. aeruginosa* Z25.1 (**b**) and treating them with PURE_G4_OEI_48_ (2.07 mg/mL) with and without Nisin Z, amlodipine (0.16 mg/mL) with and without Nisin Z, and Nisin Z (0.2 mg/mL). Positive control: infected larvae + NaCl 0.9% (grey bar); negative control: uninfected larvae + NaCl 0.9%/each treatment. The *P. aeruginosa* control corresponded to zero larvae alive at 96 h. Data are shown as the mean ± standard error of three independent determinations for 10 animals per treatment. ns, not significant; **, *p* < 0.01; ***, *p* < 0.001; ****, *p* < 0.0001 (one-way ANOVA, Tukey’s multiple comparison test). NZ, Nisin Z; PURE, PURE_G4_OEI_48_; AML, amlodipine.

Figure 4 shows the larvae’s survival rate at 96 h after being challenged with bacteria and treated with the drugs under evaluation.

After 96 h, the treatment with Nisin Z of the larvae infected with *S. aureus* significantly improved the survival rates in comparison with the control group (*p* = 0.0001) (Figure 4a). After 96 h, the larvae infected with *P. aeruginosa* showed low survival rates. However, the survival rates were significantly higher in the groups treated with Nisin Z (*p* = 0.005), with amlodipine supplemented with Nisin Z (*p* = 0.005), with PURE_G4_OEI_48_ (*p* = 0.0001), and with PURE_G4_OEI_48_ supplemented with Nisin Z (*p* < 0.0001) than that in the control group (Figure 4b).

It is worth noting that the absolute survival rates for the *P. aeruginosa* infections remained relatively low (Figure 3c,d; Figure 4b). This may be attributed to two factors: the pathogenicity of this specific *P. aeruginosa* strain, as just 100 CFU was sufficient to kill all the larvae within 24 to 48 h post-inoculation, and the difficulty in eradicating *P. aeruginosa* using these antimicrobials [35,36].

## 3. Discussion

The insect *G. mellonella* has been widely used in the last few decades as a suitable model for infection studies; for the assessment of the efficacy of antibacterial agents, such as Nisin A [37,38,39,40,41]; for the evaluation of the toxicity of several compounds [42,43,44]; and as a model host for human pathogens [45,46]. Its immune system has remarkable similarities with the one of mammals, and results from studies performed in *G. mellonella* correlate positively with other models [43,47,48]. This model complies with the principle of the 3Rs in animal experimentation and should be used prior to preclinical studies in mammal models [42,43,44]. However, the use of other animal models would also be advantageous, which represented a limitation of this study. Also, the absence of additional control groups using established antibiotics and the lack of a quantitative assessment of bacterial burden in the larvae at different time points to correlate with survival data were drawbacks of this study and should be considered in the future.

To our best knowledge, this was the first time that the activity of Nisin Z, PURE_G4_OEI_48_, and amlodipine was tested in *G. mellonella* to assess the in vivo antimicrobial activities of these compounds against infections by *S. aureus* and *P. aeruginosa* isolated from the same DFI. The health index score obtained in *G. mellonella* confirmed a positive correlation between cocoon formation and larvae health, as the larvae with a low survival rate were less likely to form cocoons. In fact, a higher activity and higher cocoon-forming ability are regularly associated with a healthier wax worm [34].

Nisin Z previously showed an increased antibacterial effect against Gram-negative bacteria when supplemented with EDTA [26], including against *P. aeruginosa* when tested in vitro [15]. However, the lack of improvement of Nisin Z antimicrobial activity against *P. aeruginosa* Z25.1 after EDTA supplementation observed in our study suggests that EDTA may not be an effective chelating agent in larvae. The removal of magnesium and calcium ions from the outer cell wall of Gram-negative bacteria promoted by EDTA, which enhances the efficacy of other antimicrobials [49], was likely inhibited in the larvae. Therefore, in the following assays, Nisin Z was tested without EDTA.

Full larval survival was achieved in those infected with *S. aureus* and treated with Nisin Z. When supplemented with Nisin Z, both PURE_G4_OEI_48_ and amlodipine showed significantly lower antibacterial activity against *S. aureus* than Nisin Z alone, suggesting that this AMP may reduce the antibacterial effectiveness of the other two drugs tested. In contrast, Nisin Z showed no antibacterial activity against *P. aeruginosa*, probably due to the presence of a double membrane layer in this Gram-negative species, which may constrain Nisin Z’s action.

The MIC values of PURE_G4_OEI_48_ determined in previous studies were generally lower [18] than the one observed in the present study. After treatment with PURE_G4_OEI_48_, larvae infected with *P. aeruginosa* showed a small but significant increase in survival. This result agrees with the results from the in vitro MIC determination, suggesting that PURE_G4_OEI_48_ was more effective against this bacterium. *P. aeruginosa*’s resilience stems from its low cell permeability, the presence of efficient efflux systems, the expression of intrinsic and acquired antibiotic resistance mechanisms, the production of virulence factors, and the expression of stress-resistant mechanisms, making it extremely difficult to eliminate [35,36]. Taking these facts into account, the low but significant *G. mellonella* survival rates after the challenge with *P. aeruginosa* and treatment with PURE_G4_OEI_48_ are very encouraging results. However, given the high dose of PURE_G4_OEI_48_ injected into the larvae (0.083mg/g), which was 10× higher than the dose of Nisin Z and 13× higher than the dose of amlodipine, it is possible that the administration of the PURE_G4_OEI_48_ antimicrobial concentrations would cause side effects in mammals. Interestingly, in our study, the administration of PURE_G4_OEI_48_ seemed to interfere with the cocoon formation process, suggesting that PURE_G4_OEI_48_ may be detrimental to larvae’s health, although its effect on mammals is unknown.

The survival of larvae infected with *S. aureus* decreased after treatment with PURE_G4_OEI_48_ and amlodipine, suggesting potential toxicity. Although these drugs do not affect larval survival when administered to non-infected larvae, they seem to interfere with the immunity of the larvae challenged with *S. aureus*, improving bacterial colonization and infection capacities. This effect was not observed in larvae infected with *P. aeruginosa*, suggesting a pathogen-specific interaction. This differential effect may be attributed to the reliance of *S. aureus* on the evasion of phagocytic clearance as a central component of its pathogenicity [50]. If the compounds interfere with host phagocytic activity, this could favor *S. aureus* survival and proliferation, whereas *P. aeruginosa*, which employs alternative virulence mechanisms, may be less affected [51]. Despite the high PURE_G4_OEI_48_ MIC values, the amlodipine MIC values obtained in our study were consistent with previous ones [19,21], suggesting that its toxicity cannot be explained by the MIC alone.

The fact that no differences in survival were detected in the larvae infected with *P. aeruginosa* after the treatment with amlodipine may be explained by the absence of true macrophages in larvae. In mammals, it is hypothesized that amlodipine concentrates within the macrophage, helping to eliminate bacteria [21]. However, *G. mellonella*’s cellular immunity relies on hemocytes, which are phagocytic cells similar to mammalian blood cells found in hemolymph and various tissues [44,52], which may explain the lack of antibacterial efficacy of amlodipine.

Future research should focus on further characterizing PURE_G4_OEI_48_ and amlodipine. Investigating their ability to permeabilize membranes using sensitive probes, as well as assessing their antimicrobial efficacy against a broader range of bacterial strains, will be essential for elucidating their mechanism of action and evaluating their potential toxicity.

## 4. Materials and Methods

### 4.1. Bacterial Strains and Cultural Conditions

The biofilm-producing DFI strains used in this study, *S. aureus* Z25.2 and *P. aeruginosa* Z25.1, were co-isolated from the same diabetic foot ulcer and fully characterized by us in previous studies [53,54,55,56], in which the bacteriostatic and antibiofilm efficacy of Nisin Z and its toxicity were determined in vitro [15]. Before testing, bacterial strains were inoculated in Brain Heart Infusion (BHI) agar (VWR, Leuven, Belgium) and incubated at 37 °C for 24 h.

### 4.2. Preparation of Solutions

Nisin Z was prepared as described [23]. Stock solutions of 0.02 g/mL from ultrapure Nisin Z (≥95% purity, NISIN Z) (Handary, Brussels, Belgium) were prepared in Milli-Q purified water (Sigma-Aldrich, Darmstadt, Germany), filtered using a 0.2 µm Millipore filter (VWR, Leuven, Belgium), and stored at 4 °C. The highest concentration tested was 0.4 mg/mL, as higher concentrations approached the solubility limit of Nisin Z in Milli-Q purified water at a neutral pH. EDTA was added to a final concentration of 0.4% (4000 µg/mL), as previously described [15].

PUREG_4O_EI_48_ was synthesized and prepared as previously indicated [18]. Then, stock solutions (33.12 mg/mL) were dissolved in Milli-Q purified water (Sigma-Aldrich, Darmstadt, Germany) and kept at 4 °C.

Amlodipine besylate (Supelco, Darmstadt, Germany) was dissolved in DMSO in a 10 mg/mL solution and kept at −20 °C. Testing solutions were prepared with Milli-Q purified water (Sigma-Aldrich, Darmstadt, Germany) and kept in the refrigerator until use.

**Figure 5:**
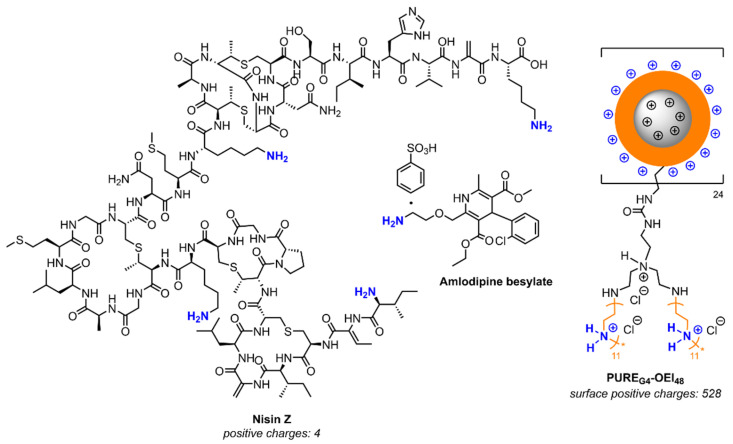
Chemical structures of Nisin Z, amlodipine, and the core–shell polycationic polyurea pharmadendrimer PURE_G4_OEI_48_ (cartoon showing only the surface structure of one branch).

The chemical structures of Nisin Z, amlodipine, and PUREG_4O_EI_48_ are depicted in Figure 5.

### 4.3. Minimum Inhibitory Concentration (MIC) of PURE_G4_OEI_48_ and Amlodipine

The MIC values of Nisin Z, PURE_G4_OEI_48_, and amlodipine toward *S. aureus* Z25.2 and *P. aeruginosa* Z25.1 DFI strains were determined as previously described [23] using a microtiter broth dilution method [57]. First, bacterial suspensions were prepared in Mueller Hinton broth, and their concentrations were adjusted to 1 × 10^6^ CFU/mL. These suspensions were placed on a 96-well flat-bottomed microtiter plate (VWR, Radnor, USA), after which four-fold serial dilutions of PURE_G4_OEI_48_ or amlodipine were added to each well (1:4 dilution). The MIC values of PURE_G4_OEI_48_ and amlodipine solutions supplemented with Nisin Z were also determined. Positive control (200 µL of bacterial suspension) and negative control wells (200 µL of broth medium plus tested compounds, with or without Nisin Z) were included in the assays. Microplates were incubated overnight at 37 °C, and MIC (mg/mL) was determined as the lowest concentration of each solution that visually inhibited microbial growth (no solution turbidity on direct observation). Three independent determinations with three technical replicates were performed on different days.

### 4.4. Galleria Mellonella Killing Assay and Quantification of S. aureus and P. aeruginosa CFU

*G. mellonella* wax moth larvae, which were previously reared in the lab at 25 °C in the dark from egg to last-instar larvae and fed with a natural diet (beeswax and pollen grains), were generously provided by Dalila Mil-Homens. Worms of the final-instar larval stage, which weighed 250 ± 25 mg, were selected for the experiments. The larvae were randomly distributed by groups (test and control groups), with each group being formed by ten healthy *G. mellonella* larvae with similar weights.

The protocol for the *G. mellonella* survival experiment was adapted from previous studies with small changes [58,59]. Briefly, DFI *S. aureus* and *P. aeruginosa* isolates were grown overnight in LB. Then, the bacterial suspensions were washed three times with 0.9% saline. After, the density of the bacterial suspensions was adjusted to 1 × 10^6^ CFU for *S. aureus* suspensions and to 100 CFU for *P. aeruginosa* suspensions (previously determined as the lethal dose), and larvae were infected with a lethal dose of both bacterial strains using a hypodermic syringe. The larvae were injected with 5 µL of each bacterial suspension via the hindmost left proleg, which was previously surface sanitized with 70% (*v*/*v*) alcohol. After approximately 1 h, the larvae were injected with 10 µL of Nisin Z (0.008 mg/g), PURE_G4_OEI_48_ (0.083 mg/g), or amlodipine (0.006 mg/g) in the hindmost right proleg. A 10 µL injection was preferred over 5 µL to increase the drug’s efficacy without compromising the larvae health. Infected larvae (5 µL each bacterial suspension) + NaCl 0.9% (10 µL) were used as positive controls. Negative controls were also included in all experiments as follows: NaCl 0.9% (5 µL) + NaCl 0.9% (10 µL) to monitor the killing due to injection trauma, as well as NaCl 0.9% (5 µL) + Nisin Z (10 µL), NaCl 0.9% (5 µL) + PURE_G4_OEI_48_ (10 µL), and NaCl 0.9% (5 µL) + amlodipine (10 µL).

After the injections, the larvae were kept in Petri dishes and maintained in the dark at 37 °C for 96 h. The larval survival was assessed daily during this period, and caterpillars were considered dead based on their lack of mobility in response to touch. Each larva was observed every day based on the *G. mellonella* health index, which scores four main parameters: larval survival, melanization, mobility, and cocoon formation, as described in [34]. Three independent experiments were performed on different days, using a total of 30 larvae.

To determine the CFU of the bacterial suspensions injected in the larvae, the suspensions were serially diluted in 0.9% saline and plated on BHI agar. The bacterial colonies were counted after incubation at 37 °C for 24 h.

### 4.5. Galleria Mellonella Toxicity Assay

Nisin Z, PURE_G4_OEI_48_, and amlodipine toxicity were first evaluated. The *G. mellonella* killing assay was based on the above descriptions. The larvae were first injected with 5 µL of 0.9% saline at the hindmost left proleg and then with 10 µL of each drug at the hindmost right proleg. The larvae’s survival was assessed daily during a period of 96 h. A control group [NaCl 0.9% (5 µL) + NaCl 0.9% (10 µL)] was also included in the assay. Three independent experiments were performed on different days (a total of 30 larvae).

### 4.6. Statistics

Data were analyzed using GraphPad Prism software for Windows version 9.4.1 (GraphPad, San Diego, CA, USA) and shown as the mean ± standard error. One-way ANOVA (*p* < 0.0001) and Tukey’s multiple comparisons test were performed. Kaplan–Meier survival curves were used for *G. mellonella* with the Mantel–Cox test significance between survival curves of treated larvae and untreated larvae.

## 5. Conclusions

Pre-screen *G. mellonella* experiments showed that Nisin Z exhibited full anti-staphylococcal activity in vivo, in agreement with the low MIC values observed in vitro. Although the larvae survival rates were low, the PURE_G4_OEI_48_ treatment statistically increased the survival of those infected with *P. aeruginosa*, partially corroborating the in vitro findings, where PURE_G4_OEI_48_ showed moderate antimicrobial activity against this strain. In contrast, amlodipine showed no inhibitory activity against *S. aureus* or *P. aeruginosa* in vivo, which was inconsistent with its low MIC values. The results obtained in this study are significant, suggesting that Nisin Z and PURE_G4_OEI_48_ could be individually used as topical coadjuvants in the treatment of mild superficial DFIs. However, further studies are needed to evaluate PURE_G4_OEI_48_ toxicity and efficacy in more complex models.

## Figures and Tables

**Table 1:** Minimum inhibitory concentrations (MICs) of Nisin Z, PURE_G4_OEI_48_, and amlodipine toward *S. aureus* Z25.2 and *P. aeruginosa* Z25.1.

Strains	MIC mg/mL (µM ^1^)		
	Nisin Z	PURE_G4_OEI_48_	Amlodipine
*S. aureus* Z25.2	0.01 (3)	2.07 (40.5)	0.04 (97.8)
*P. aeruginosa* Z25.1	>0.4 ^2^ (>120)	0.52 (10.2)	0.16 (391.2)

^1^ The MIC values are primarily reported in mg/mL for direct comparison of antimicrobial efficacy. Corresponding µM values are presented only to emphasize the differences in molecular mass between the tested compounds. ^2^ The exact MIC could not be determined within the concentration range tested.

## Data Availability

The data presented in this study are contained within this article.

## Abbreviations

The following abbreviations are used in this manuscript:

AMPs	Antimicrobial peptides
BHI	Brain heart infusion
CFU	Colony forming units
DFIs	Diabetic foot infections
DFUs	Diabetic foot ulcers
MIC	Minimum inhibitory concentration
SMAMPs	Synthetic mimics of antimicrobial peptides
